# Supplementary material for: Causal interplay between lactose intolerance and gut microbiota: a combined bidirectional Mendelian randomization and in vivo validation study
Source: Front Nutr. 2026 Jun 1;13:1803337. doi: 10.3389/fnut.2026.1803337 (PMC13265576; doi:10.3389/fnut.2026.1803337)
Supplement: Supplementary file 1 [file Data_Sheet_1.zip › supplementary materials/Forward/leave one out cross validation/ebi-a-GCST90027788.finngen_R12_E4_LACTONAS.pdf]

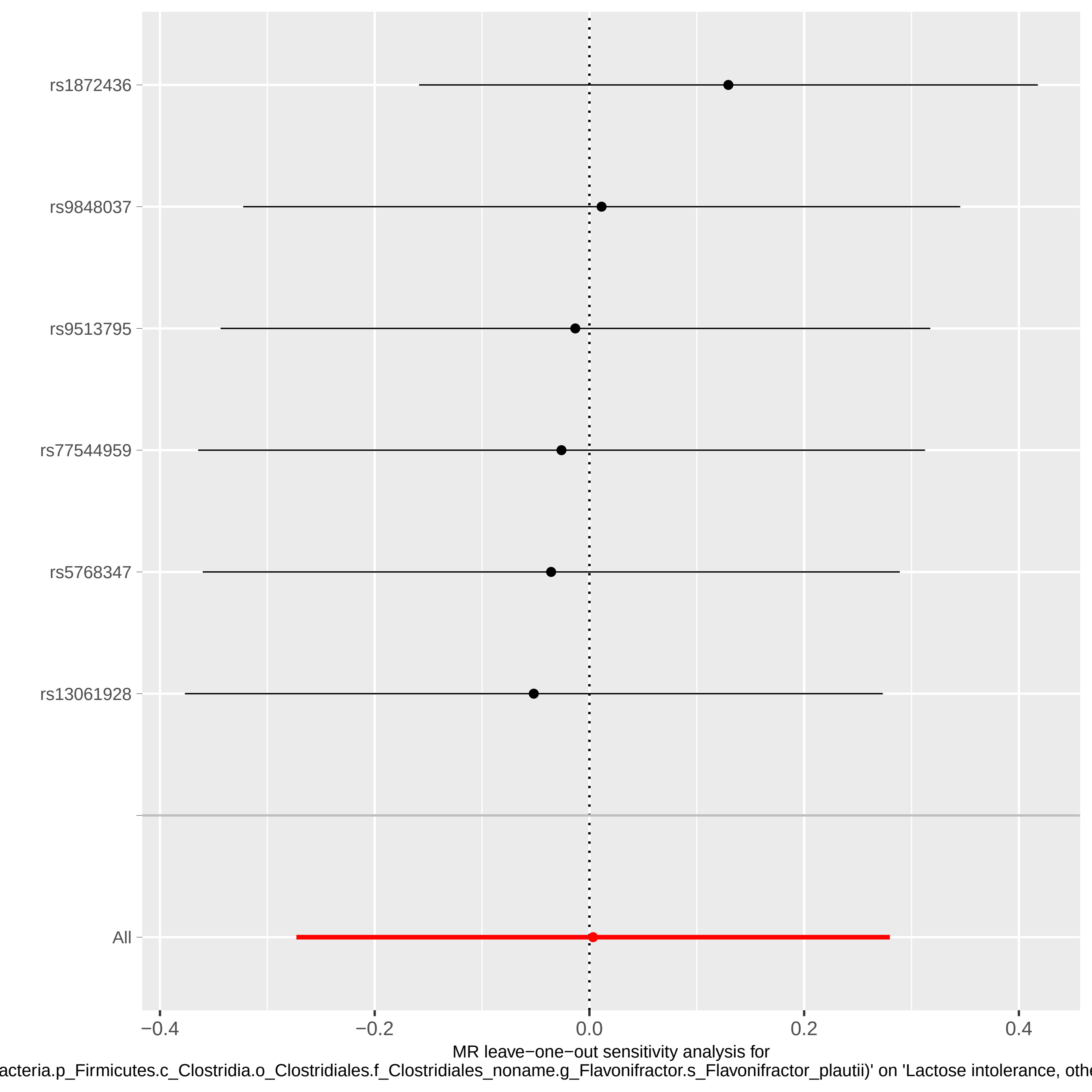
rs1872436
rs9848037
rs9513795
rs77544959
rs5768347
rs13061928
All
−0.4
−0.2
0.0
0.2
0.4
MR leave−one−out sensitivity analysis for
acteria.p_Firmicutes.c_Clostridia.o_Clostridiales.f_Clostridiales_noname.g_Flavonifractor.s_Flavonifractor_plautii)' on 'Lactose intolerance, othe
